# New Insights on Heme Uptake in *Leishmania* spp.

**DOI:** 10.3390/ijms231810501

**Published:** 2022-09-10

**Authors:** María Cabello-Donayre, Lina M. Orrego, Elisa Herráez, Raquel García-Hernández, José M. Pérez-Victoria

**Affiliations:** 1 Instituto de Parasitología y Biomedicina “López-Neyra”, Consejo Superior de Investigaciones Científicas, PTS Granada, 18016 Granada, Spain; 2 Experimental Hepatology and Drug Targeting, Instituto de Investigación Biomédica de Salamanca, University of Salamanca, 37007 Salamanca, Spain; 3 Center for the Study of Liver and Gastrointestinal Diseases, Carlos III National Institute of Health, 28029 Madrid, Spain

**Keywords:** heme, porphyrin trafficking, *Leishmania*, protozoan parasites, neglected diseases

## Abstract

The protozoan parasite *Leishmania*, responsible for leishmaniasis, is one of the few aerobic organisms that cannot synthesize the essential molecule heme. Therefore, it has developed specialized pathways to scavenge it from its host. In recent years, some proteins involved in the import of heme, such as *L*HR1 and *L*FLVCRB, have been identified, but relevant aspects regarding the process remain unknown. Here, we characterized the kinetics of the uptake of the heme analogue Zn(II) Mesoporphyrin IX (ZnMP) in *Leishmania major* promastigotes as a model of a parasite causing cutaneous leishmaniasis with special focus on the force that drives the process. We found that ZnMP uptake is an active, inducible, and pH-dependent process that does not require a plasma membrane proton gradient but requires the presence of the monovalent cations Na^+^ and/or K^+^. In addition, we demonstrated that this parasite can efflux this porphyrin against a concentration gradient. We also found that ZnMP uptake differs among different dermotropic or viscerotropic *Leishmania* species and does not correlate with *L*HR1 or *L*FLVCRB expression levels. Finally, we showed that these transporters have only partially overlapping functions. Altogether, these findings contribute to a deeper understanding of an important process in the biology of this parasite.

## 1. Introduction

Leishmaniasis is a parasitic disease transmitted by the bite of an infected female sandfly. At least 20 *Leishmania* species cause human infection, leading to a wide spectrum of clinical manifestations ranging from mild cutaneous and disfiguring mucocutaneous forms to a more severe visceral leishmaniasis form which is often fatal if untreated [1]. Currently, there is a limited arsenal of leishmanicidal drugs that are far from ideal since they frequently induce toxic side effects and present a low effectivity associated with growing therapeutic failure [2]; therefore, the discovery and development of new pharmacological compounds are urgently needed. A rational way to identify promising therapeutic targets is to exploit the biochemical differences between parasites and their human host. One difference involves heme metabolism, which has been proposed as a target for new drugs against protozoan parasites [3]. Heme, an iron-containing porphyrin, is an essential cofactor of proteins involved in crucial biological processes, including oxygen transport and storage, mitochondrial electron transfer, signal transduction, drug metabolism, microRNA processing, and regulation of antioxidant-defense enzymes [4]. Trypanosomatid parasites are among the few organisms that, even though they depend on oxidative phosphorylation, are unable to synthesize heme and must, therefore, scavenge this molecule from their host to fulfill their heme requirements [5]. Uncovering the mechanisms that allow *Leishmania* to capture this molecule could help the development of novel therapeutic strategies to fight leishmaniasis.

*Leishmania* can obtain heme via two independent routes (review in [6]): (i) clathrin-dependent endocytosis of hemoglobin (Hb) mediated by the HbR receptor located in the flagellar pocket, which allows rapid Hb internalization in a Rab5b- and Rab7-regulated process and degradation in lysosomes to generate intracellular heme, which is subsequently exported to the cytosol via *L*HR1 [7,8,9,10,11,12,13]; and (ii) direct heme transport via the plasma membrane [14,15]. This entry of free porphyrins was characterized in the viscerotropic species *Leishmania donovani* and was shown to be protein- and temperature-dependent [16]. The first protein responsible for this transport was discovered in the cutaneous species *Leishmania amazonensis* and named *L*HR1 (*Leishmania* Heme Response 1) due to its homology with the membrane protein HRG4 (Heme-Response Gene 4) of *Caenorhabditis elegans* involved in heme uptake [14]. *L*HR1 is located at the plasma membrane of both promastigote and intracellular amastigote forms of *Leishmania*, where it participates in the uptake of exogenous heme [14], and is also found in endolysosomal compartment membranes, where it mediates the import of Hb-derived free heme into the cytosol [12]. *L*HR1 is essential, as CRISPR/Cas9-mediated null mutants are only obtained in the presence of episomal copies of the gene (MCD and JMPV, manuscript in preparation). In addition, *L*HR1 is required for parasite virulence, since single knockout parasites are unable to replicate intracellularly in macrophages and show a severe defect in the development of cutaneous lesions in mice [17]. A second protein involved in heme uptake was subsequently identified in the cutaneous species *Leishmania major*. This transporter, named *L*FLVCRB, could be orthologous to the mammalian heme transporter FLVCR2, it is located at the plasma membrane of parasites and participates in the uptake of labile heme [15]. Similar to *L*HR1, *L*FLVCRB is also essential as CRISPR/Cas9-mediated knockout parasites were only obtained in the presence of an episomal copy of the gene [15]. Additionally, the deletion of one allele of the *L*FLVCRB gene affects the intracellular replication of parasites in infected macrophages and hampers their virulence in a mouse infection assay [15].

Despite all these advances in our understanding of heme uptake by these parasites, many relevant mechanistic aspects remain unknown. For example, the driving force that guides porphyrin transport through the parasite’s plasma membrane has not been identified, nor has whether there are differences between *Leishmania* species depending on whether they are viscerotropic or dermotropic. Access to heme in the infected hosts of these two types of parasites is probably very different and, in fact, porphyrin uptake in *L. donovani* seems to be more efficient than in *L. amazonensis* [14,16]. In this context, a deeper understanding of the heme uptake process in different *Leishmania* species is required to improve our knowledge about the biology of these organisms. In the present work, we biochemically characterized the uptake (and efflux) of the fluorescent heme analogue ZnMP in *L. major* as a model of a parasite causing cutaneous leishmaniasis, with special focus on the force driving the process. We also analyzed ZbMP uptake in different *Leishmania* species to determine the possible effect of their host tropism (dermotropic or viscerotropic) on the uptake process. Finally, *L*HR1 and *L*FLVCRB gene expression levels in these *Leishmania* species grown under different heme availability conditions and the correlation between ZnMP uptake ability and the expression levels of these genes were analyzed.

## 2. Materials and Methods

### 2.1. Chemical Compounds

Dibasic potassium phosphate, monopotassium phosphate, sodium phosphate, sodium chloride, potassium chloride, magnesium chloride, calcium chloride, choline, D-(+)-glucose, HEPES, Hb, hemin, and budesonide were obtained from Sigma-Aldrich (San Luis, Misuri, Estados Unidos). ZnMP was from Frontier Scientific, and Sytox-green was from Molecular Probes (Invitrogen, Waltham, Massachusetts, Estados Unidos). The purity of compounds, determined by thin-layer chromatography, was higher than 98%. All reagents were of analytical grade.

### 2.2. Parasite Strains and Culture Conditions 

*Leishmania major* MHOM/IL/80/Friedlin, *L. donovani* HU3, *Leishmania mexicana* MNYC/BZ/B2 M379, *Leishmania infantum* JPCM5, and *Leishmania tarentolae* Parrot-TarII were cultured as axenic promastigotes at 28 °C in modified RPMI-1640 medium (Invitrogen, Carlsbad, CA, USA) supplemented with 10% heat-inactivated fetal bovine serum (FBS, Invitrogen) as described in [18]. Heme-depleted parasites were obtained and verified as described in [19]. Briefly, promastigotes were cultured for 24 h under the same conditions but substituting FBS for 20% heme-depleted FBS (hdFBS), prepared as previously described [12,20], supplemented or not with heme or Hb.

### 2.3. LHR1 and LFLVCRB Expression Analysis Using Quantitative Real-Time PCR 

Parasites of each strain were grown in modified RPMI-1640 medium containing 10% FBS or 20% hdFBS supplemented or not with heme. Total RNA extraction from 3 × 10^7^ promastigotes was performed using the RNeasy Mini Kit (QIAGEN, Beverly, MA, USA) and subsequently treated with TURBO DNase (Invitrogen) to eliminate contaminating DNA according to the manufacturer’s instructions. cDNA from *Leishmania* strains was synthesized from 1 µg of total RNA using the qSCRIPT^TM^ cDNA Synthesis kit (Quanta Biosciences, Inc. Hilden, Germany) according to the manufacturer’s instructions. To determine the mRNA levels of the *L*HR1 and *L*FLVCRB genes, species-specific primers for RT-qPCR were designed using Primer3 [21]. LmjF.04.0930 and the homologous genes for each species were used as housekeeping genes since, contrary to *GADPH*, their expression in *L. major* parasites is not altered in response to heme (MCD, LOZ, RGH and JMPV, manuscript in preparation) (accession codes for each gene in Supplementary Material, Table S2). Standard curves for each primer pair were generated with two-fold serial dilutions of the synthetized cDNA to determine primer efficiency. The specificity of the amplification was verified via melting curve analysis according to the BioRad (Hercules, CA, USA) RT-PCR applications guide. Quantitative real-time PCR was performed using Perfecta SYBR Green Supermix (Quanta Bioscience), and relative gene expression data analysis was performed using the 2^−^^∆∆CT^ Method [22], as described in [19].

### 2.4. ZnMP Uptake and Efflux by Leishmania Promastigotes 

Uptake of the fluorescent heme analog ZnMP was measured with flow cytometry, as previously described [12], or with spectrofluorimetry. Briefly, for the first case, 1 × 10^7^ promastigotes in their exponential growth phase, cultured in modified RPMI-1640 medium containing 10% FBS (complete medium) or 20% hdFBS supplemented or not with heme or Hb, were washed with phosphate-buffered saline (PBS), resuspended in HPMI medium (20 mM HEPES pH 7.25, 132 mM NaCl, 3.5 mM KCl, 0.5 mM MgCl_2_, 1 mM CaCl_2_, 5 mM glucose), and incubated at 28 °C for 2 h. At this point, 10 µM ZnMP was added to the medium and incubated for 10 min at 28 °C or 4 °C. Parasites were centrifuged at 14,500× *g* for 30 s at 4 °C and washed for 10 min with ice-cold 5% BSA in PBS to remove porphyrin nonspecifically bound to the plasma membrane (back-exchange step). To verify that promastigotes remain healthy after 2 h incubation in HPMI medium and 10 min incubation with 10 µM ZnMP, cell viability was assessed via flow cytometry using 20 nM SYTOX^TM^ Green (Invitrogen) according to manufacturer’s instructions. For biosafety reasons, parasites were fixed with 5% paraformaldehyde. A total of 10^5^ cells was acquired and analyzed via flow cytometry using a FacsAria Cell Sorter III (Becton Dickinson, SanJose, CA, USA) (excitation at 405 nm and emission between 575 and 585 nm). The net uptake values plotted are the differences between values obtained at 28 °C and 4 °C in order to decrease the contribution of non-specific association of ZnMP to the parasite surface. As pH influences the ZnMP fluorescence measurement (Supplementary Figure S1), ZnMP uptake at different pH values was analyzed using spectrofluorimetry. Concisely, parasites were lysed with five freeze–thaw cycles and resuspended in 100 mM phosphate buffer pH 8.3, including 1% Triton X-100. Fluorescence was determined using an Aminco-Bowman Series 2 Luminescence Spectrometer (SLM-AMINCO) by exciting the samples at 402 nm and collecting the fluorescence between 550 and 600 nm. The relative fluorescence (measured in relative fluorescence units (RFU)) was normalized against total protein amount. Protein quantification was performed via the Bradford method [23] using a BSA standard curve containing the same concentration of Triton X-100 present in lysis buffer. The ionophores FCCP (H^+^), valinomycin (K^+^), and monensin (Na^+^) were used in conjunction with ZnMP for transport assay at 10 µM, 200 µM, and 50 µM, respectively, as described in [24]. For experiments involving respiratory chain inhibitors, parasites were pre-incubated for 30 min with the inhibitors (1 µM oligomycin A, 400 µM KCN, 0.5 µM antimycin A, and 10 µM rotenone) prior to porphyrin uptake assay, as described in [25]. Cyclosporin A and verapamil, ABC transporter inhibitors, were added at 10 µM at the time of porphyrin uptake assay, as described in [26]. To determine the temperature dependence of porphyrin uptake, *L. major* promastigotes were incubated with 200 μM porphyrin (50 μM ZnMP/150 μM hemin) at both 4 °C and 28 °C, in a time course experiment and analyzed using flow cytometry. For the ZnMP efflux assay, after ZnMP incubation as described above, parasites were resuspended in HPMI medium at 28 °C or 4 °C, and samples were collected at different time points for analysis. Collected parasites were centrifuged to separate the pellet and supernatant fractions, and intracellular and extracellular ZnMP contents were analyzed via flow cytometry and spectrometry, respectively. To evaluate ZnMP efflux in the presence of high concentrations of extracellular hemin, parasites were incubated for 10 min at 28 °C in the presence of increasing hemin concentrations after ZnMP uptake and, subsequently, intracellular ZnMP was measured by flow cytometry.

### 2.5. Evaluation of LFLVCRB and LHR1 Gene Essentiality Using CRISPR-Cas9 

To test the essentiality of the *L*FLVCRB and *L*HR1 genes, the open reading frame of both genes was deleted from the *L. major* genome using the CRISPR-Cas9 tool kit described previously [27] and kindly provided by Dr. Eva Gluenz (University of Oxford, UK). Log-phase promastigotes of *L. major* Cas9/T7 [19] expressing or not an episomal copy of *L*FLVCRB or *L*HR1 (amplified using cd1/cd2 for *L*FLVCRB [15] and P13/P14 for *L*HR1 [12]), were co-transfected with donor DNA and single-guide RNA (sgRNA) templates using program V-033 of the Amaxa Nucleofector System (Lonza). Deleted genes were replaced by donor DNAs containing a puromycin resistance cassette flanked by 30-nt homology arms from 5′UTR and 3′UTR specific sequences of the *L*FLVCRB and *L*HR1 genes, which were amplified from the pT Puro plasmid using primer pairs cd17/cd18 and cd56/cd57 [15], respectively. sgRNA templates were amplified using the common reverse primer G00 (sgRNA scaffold) and specific forward primers for 5′sgRNA and 3′ sgRNA of both genes (cd19 and cd20 for *L*FLVCRB and cd58 and cd59 for *L*HR1, taken from [15]), which contained a sequence encoding the T7 promoter for in vivo transcription. Primers were designed using an online resource (http://www.leishgedit.net/Home.html accessed on 1 Octubre 2018). Transfected parasites were plated onto semi-solid culture medium plus 20% FBS and 30 µg/mL puromycin, and selected clones were propagated in liquid modified RPMI-1640 medium supplemented with 10% FBS and the same concentration of puromycin. Gene deletion and puromycin cassette integration were confirmed via PCR analysis using specific primers for each gene (Supplementary Material, Table S1) as described in [15].

### 2.6. HPLC-MS/MS Analysis of Hemin 

To evaluate the effect of extracellular ions present in the uptake incubation buffer, intracellular hemin was quantified by liquid chromatography–electrospray tandem mass spectrometry via an adaptation of a previously published method [28], as described in [15].

### 2.7. Statistical Analysis

Experiments were performed three times in duplicate. All data are presented as the mean, and error represents S.E.M. Statistical significance was determined using Student’s *t*-test. *p* ≤ 0.05 (* *p* ≤ 0.05, ** *p* ≤ 0.005, *** *p* ≤ 0.0005) was considered statistically significant with respect to the groups indicated in each figure.

## 3. Results and Discussion

### 3.1. Kinetics of ZnMP Uptake in L. major Promastigotes

As heme availability in the infected host probably differs between viscerotropic and dermatropic species of *Leishmania*, the mechanisms developed by the parasites for heme import could have some differences. Heme uptake has already been biochemically characterized in the viscerotropic species *L. donovani* [16]. To evaluate this process in a species causing cutaneous leishmaniasis, we have used *L. major* promastigotes and *Zn*(II) *Mesoporphyrin* IX (ZnMP), a natural zinc (II) porphyrin derivative previously used as a fluorescent heme analogue in different cell lines [29,30,31]. Previous studies demonstrated the usefulness of fluorescent heme analogues in characterizing the mechanism of heme internalization in hepatocytes and in intestinal cells, in mammals [29,32], and in parasites such as *T. cruzi* [26], *L. amazonensis* [14], *L. donovani* [16], and *L. major* [12,15]. 

**Figure 1:**
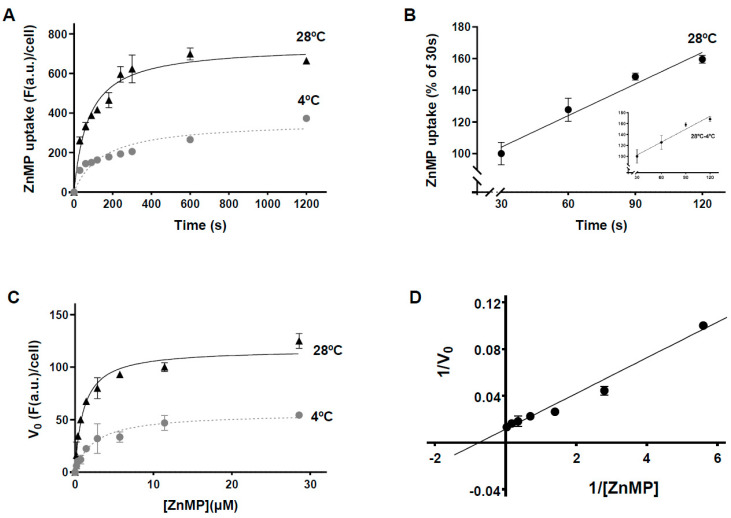
Kinetic characterization of the ZnMP uptake process in *L. major* promastigotes. (**A**) Temperature and time dependence of ZnMP incorporation. The import of 200 μM porphyrin (50 μM ZnMP/150 μM hemin) was measured as described in Materials and Methods at 28 °C (black) or 4 °C (gray) over 20 min. (**B**) ZnMP uptake follows a linear function during the first 120 s. Data obtained in A during the first 120 s at 28 °C were adjusted to a linear function (R^2^ = 0.968). The inset shows the net porphyrin incorporation (difference between values obtained at both temperatures) (R^2^ = 0.965). (**C**) ZnMP uptake by *L. major* is substrate saturable. Initial porphyrin uptake (V_o_) was measured at the ZnMP concentrations indicated over 120 s at 28 °C (black) or 4 °C (gray) as described in Materials and Methods. The data obtained were adjusted to a classic Michaelis–Menten hyperbolic function (R^2^ = 0.978 and R^2^ = 0.977, respectively). (**D**) Lineweaver–Burk representation of the net porphyrin uptake. (R^2^ = 0.984). Data are representative of three experiments and values expressed as mean ± standard error of the mean (SEM). **F**: Fluorescence; a.u.: arbitrary units; V_0_: initial porphyrin uptake.

For kinetic characterization of the porphyrin uptake process, the initial porphyrin concentration was set at 200 µM, a 13-fold-higher concentration than the previously established Km for *L. donovani* promastigotes [16], thus ensuring that the substrate concentration was not limiting, at least at the start of the process [25]. To prevent ZnMP toxicity, 200 µM porphyrin was obtained using 50 µM ZnMP and 150 µM hemin. No significant reduction of parasite viability was observed under these conditions using the vital dye Sytox-Green (data not shown). Then, ZnMP uptake was measured at different time points at 28 °C (the temperature at which parasites are cultured) or on ice (to greatly decrease endocytosis and transporter activity [29]). ZnMP uptake was temperature-dependent (Figure 1A) and linear during the first 120 s (Figure 1B, R^2^ = 0.968). Therefore, an incubation time of two minutes was selected for measuring the initial uptake velocity (V_0_) to determine the Km of the process.

To this end, ZnMP uptake V_0_ was measured as a function of substrate concentration at both temperatures. The data obtained at 28 °C showed a saturating uptake of porphyrin and were adjusted to a classic Michaelis–Menten hyperbolic function (R^2^ = 0.978) (Figure 1C). A saturating curve was also observed when the assay was performed on ice (Figure 1C, R^2^ = 0.977), probably due to the binding of porphyrin to its transporter(s) and/or the plasma membrane. A Km value for net porphyrin transport of 1.0 ± 0.1 µM was estimated via a Lineweaver–Burk plot of net uptake values (difference between values obtained at both temperatures) (Figure 1D, R^2^ = 0.984). 

Interestingly, the porphyrin uptake Km for *L. donovani* was more than an order of magnitude higher than that obtained for *L. major* (14.9 ± 4.5 µM vs. 1.0 ± 0.1 µM) [16]. These differences were probably not due to the use of different heme analogs (Mg-PPIX and ZnMP, respectively) since the uptake of both is competed in the same way in the presence of hemin, but probably to a greater transporter(s) affinity for porphyrins in the case of *L. major*. This could be related to the fact that both *Leishmania* species have different tissue tropisms; thus, their access to the heme group is probably different, as we discuss below.

### 3.2. ZnMP Uptake in L. major Is a pH-Dependent Process Not Requiring a Plasma Membrane Proton Gradient

It has been suggested that pH affects heme uptake in *Leishmania* parasites, as a drop in pH to 5.4 in the binding buffer elevates hemin binding by approximately five-fold [^55^Fe] in *L. amazonensis* promastigotes [33]. This might predict a more efficient heme uptake in the acidic environment in which intracellular amastigotes reside, probably similar to the transport of glucose, proline, nucleosides, and polyamines, which has optimal activity at acidic pH values in amastigotes, supporting the concept that nutrient transport systems at the intracellular stage are adapted to function effectively in the acidic environment of the parasitophorous vacuole. [34,35,36,37,38]. Therefore, we decided to evaluate the effect of extracellular pH on porphyrin uptake. Since the pH of the medium alters the fluorescence of the ZnMP probe (Supplementary Figure S1), quantification of intracellular ZnMP was performed spectrophotometrically (instead of using flow cytometry) after lysing the parasites and adjusting the pH of the different samples to 8.3.

**Figure 2:**
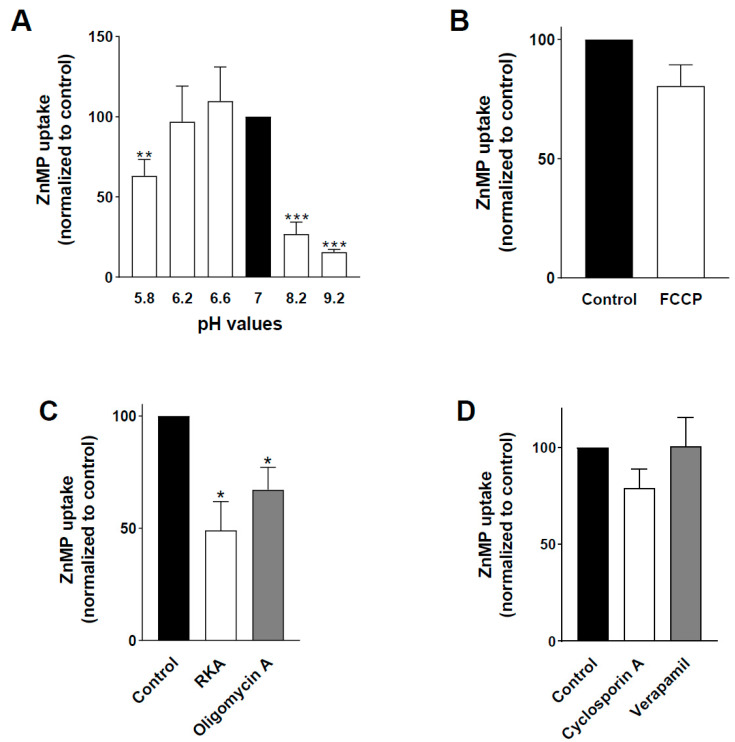

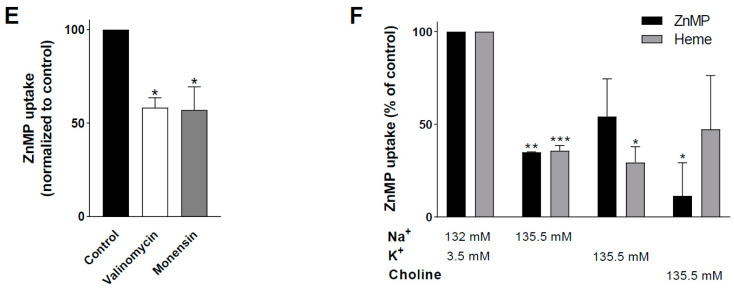
Analysis of the driving force of porphyrin import in *L. major* promastigotes. (**A**) ZnMP uptake is pH-dependent. Parasites were incubated in different pH buffers containing 10 µM ZnMP at 28 °C and 4 °C for 10 min. Intracellular ZnMP was measured via spectrofluorimetry as detailed in Materials and Methods, normalized against protein amount and net intracellular ZnMP accumulation at each pH, represented as a percentage of that found at pH 7. (**B**) ZnMP uptake does not depend on the plasma membrane proton gradient. Parasites were incubated with 10 µM ZnMP in the absence (control) or presence of 10 µM FCCP (a H^+^ ionophore) at 28 °C and 4 °C for 10 min, and net ZnMP uptake was quantified by flow cytometry as described in Materials and Methods. (**C**) ZnMP uptake is an active transport process. Parasites were pre-incubated for 30 min with the energy depleters RKA (mixture of the respiratory chain inhibitors rotenone, KCN and antimycin A, at 0.5 µM) or oligomycin A (mitochondrial F1F0 ATP synthase inhibitor, 1 µM) before analyzing ZnMP uptake as described in (**B**,**D**) ABC transporters are not involved in ZnMP transport. Parasites were incubated with 10 µM ZnMP in the absence (control) or presence of 10 µM cyclosporin A or verapamil, and the net ZnMP uptake was quantified as described above. (**E**,**F**) Porphyrin import requires monovalent ions. (**E**) Effect of K^+^ and Na^+^ ionophores on ZnMP accumulation. Parasites were incubated with 10 µM ZnMP in the absence (control) or presence of K^+^ (200 µM valinomycin) and Na^+^ (50 µM monensin) ionophores, and net ZnMP uptake was measured as described above. F. Effect of extracellular K^+^ and Na^+^ on porphyrin accumulation. Parasites were incubated with 10 µM ZnMP or hemin in uptake buffer containing (control) Na^+^ and/or K^+^ or not. Ionic strength was kept constant with choline (135.5 mM) when both ions were absent. Net ZnMP uptake was measured as described above, whereas net heme uptake was quantified by HPLC-MS/MS as described in Material and Methods. Data were represented as percentage of control uptake found. Experiments were performed three times in duplicate and data expressed as mean ± SEM. *p* ≤ 0.05 (* *p* ≤ 0.05, ** *p* ≤ 0.005, *** *p* ≤ 0.0005) was considered statistically significant with respect to the control groups indicated in each figure.

As shown in Figure 2A, ZnMP uptake in *L. major* promastigotes was a pH-dependent process that was more efficient at a pH of between 6–7, decreasing by over 70% at basic pH values (≥8). However, ZnMP uptake was also diminished in more acidic conditions (40% at pH 5.8), in contrast to the previously discussed binding results described in *L. amazonensis* [33]. Interestingly, this behavior was also observed for the *L*FLVCRB-mediated heme transport in the oocyte model, in which hemin uptake was higher at a pH of around 7 and was reduced by 90% at basic pH values and by 35% at acid pH [15].

To determine whether the pH dependence of this transport (Figure 2A) was due to the involvement of H^+^ as a driving force for the process, ZnMP uptake was measured in the presence of the H^+^ ionophore FCCP, which dissipates the plasma membrane H^+^ gradient [25,39]. Figure 2B shows that ZnMP uptake in promastigotes was not significantly altered by FCCP. A possible alternative explanation for the observed pH sensitivity of the ZnMP uptake process could be that the protonation level of the transporter, which changes with pH, affects its interaction with ZnMP or the porphyrin transport process itself.

### 3.3. ZnMP Uptake in L. major Promastigotes Is an Active Process

The temperature dependence of ZnMP uptake in *L. major* promastigotes suggested that it was an active, energy-dependent process. To confirm this, we measured ZnMP transport in the presence of different energy depleters. These compounds were added 30 min before the transport assay to deplete cell ATP, as described in [25]. The energy depleters tested were a mixture of the respiratory chain inhibitors rotenone, KCN, and antimycin A (RKA) and the mitochondrial F_0_F_1_ ATP synthase inhibitor oligomycin A. Figure 2C shows that the decrease in ATP levels produced by preincubation with these energy depleters significantly inhibited ZnMP uptake. This result suggested that porphyrin uptake through the plasma membrane is indeed carried out by an active transport process, as expected from the presence of charged carboxylates in the hydrophobic tetrapyrrole moiety [4,40]. This active ZnMP transport could be primary—i.e., coupled directly to ATP hydrolysis—or secondary—if coupled with the pro-gradient transport of another substrate—either in the same (symport) or opposite direction (antiport). The gradient of the co-transported substrate would be generated with energy expenditure.

### 3.4. ABC Transporters Inhibitors Do Not Affect L. major ZnMP Uptake

ABC transporters are examples of primary active transporters. The two heme importers proposed in *Leishmania* (*L*HR1 and *L*FLVCRB) do not belong to this superfamily. However, it was suggested that heme uptake in *T. cruzi* is carried out by these transporters, as uptake diminished in the presence of classic ABC transporter inhibitors such as cyclosporin A and verapamil [31]. In addition, ABC transporters involved in porphyrin trafficking have also been described in eukaryotes: ABCG2 [4,41] and ABCB6 in mammals [42,43], MRP-5/ABCC5 in worms [44], and LABCG5 in *Leishmania* [16], albeit working as exporters. Few eukaryote ABC transporters are known to function as importers. However, ABC transporters involved in heme uptake have been described in bacteria [45]. Therefore, we measured ZnMP uptake in the presence of cyclosporin A and verapamil. Contrary to what was described in *T. cruzi*, these compounds did not significantly affect porphyrin entry in *L. major* (Figure 2D). Consequently, heme internalization in *Leishmania* probably does not involve uptake via an ABC transporter.

### 3.5. L. major Porphyrin Uptake Requires Monovalent Ions

We then analyzed the putative involvement of secondary active transporters in ZnMP uptake. These proteins work by coupling their transport to the gradient of a second solute, typically an ion such as Na^+^, K^+^ or H^+^ [46]. As the results using FCCP (Figure 2B) suggested that protons were not involved in the process, we studied the effect of Na^+^ and K^+^ as second solutes.

Thus, we quantified ZnMP uptake in the presence of the ionophores valinomycin and monensin, which dissipate the sodium and potassium gradients, respectively. As seen in Figure 2E, ZnMP uptake decreases by about 45% when the potassium and sodium ion gradient was reduced. To confirm these results, we also quantified both ZnMP and hemin uptake in the absence of Na^+^ and K^+^ ions. Parasites exhibited a 60–80% decrease in porphyrin uptake when assayed in the absence of Na^+^ and/or K^+^ ions (Figure 2F). When both ions were absent, the ionic strength was kept constant by adding 135.5 mM choline [25,47] to avoid a possible effect of decreased ionic strength on the transport process after ion removal.

Altogether, these results suggested that porphyrin uptake by *Leishmania* is a carrier-mediated secondary active transport process that requires the presence of Na^+^ and/or K^+^ ions. The driving force regulating *L*HR1-mediated heme transport has not yet been elucidated, but *L*FLVCRB-mediated heme uptake in oocytes expressing the *L. major* transporter also depends on the presence of these monovalent ions [15]. Another alternative explanation is a putative requirement of monovalent ions to stabilize the porphyrin transporter(s) itself or the interaction between the substrate and its binding site.

### 3.6. ZnMP Internalization in L. major Promastigotes Is Induced in the Absence of Heme

**Figure 3:**
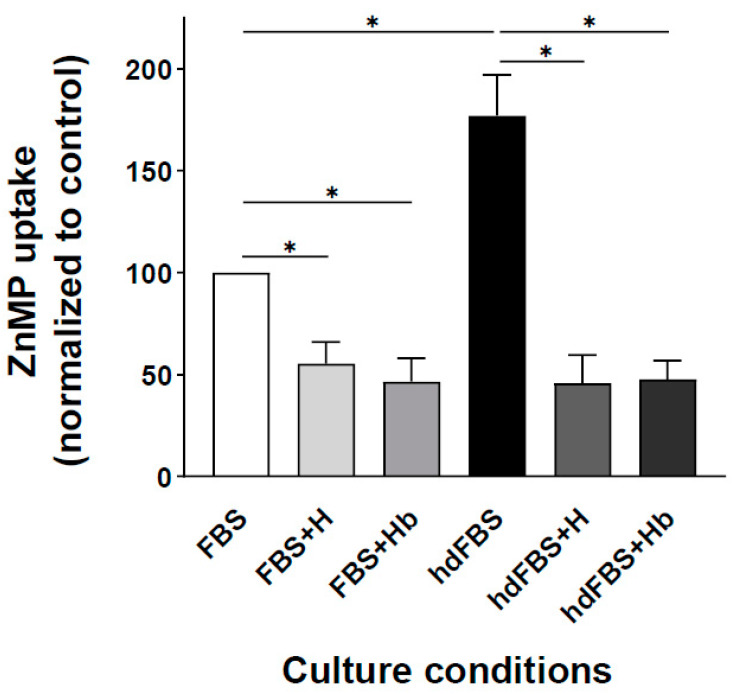
Heme deprivation induces ZnMP uptake in *L. major* promastigotes. Log-phase *L. major* promastigotes were grown in culture medium containing 10% FBS (fetal bovine serum) or 20% hdFBS (heme-depleted FBS) supplemented or not with 10 µM heme (H) or 2.5 µM hemoglobin (Hb). Net ZnMP uptake was then measured as described in Materials and Methods. Data represented as percentage uptake found after culturing the parasites in FBS-supplemented medium. Experiments were performed three times in duplicate, and data expressed as mean ± SEM. * *p* ≤ 0.05 was considered statistically significant.

Heme deprivation promotes porphyrin uptake by *L. amazonensis* and *L. infantum* promastigotes, probably because these conditions increase *L*HR1 expression [14,48]. Therefore, we next investigated how heme availability affects ZnMP uptake in *L. major* promastigotes. Parasites were grown in heme-depleted or complete culture medium, in both cases in the absence or presence of 10 µM hemin or 2.5 µM hemoglobin (equivalent to 10 µM heme). After 24 h of incubation, parasites were washed to remove excess porphyrins and resuspended in HPMI glucose medium, and ZnMP uptake was measured by flow cytometry. As shown in Figure 3, heme deprivation via medium supplemented with hdFBS increased the parasite’s ZnMP uptake by almost 80% compared with parasites grown in complete medium containing normal serum (FBS). This higher uptake decreased almost four-fold when parasites were incubated with hdFBS including 10 µM heme in the form of hemin or Hb. A similar result was observed in parasites grown in complete medium (FBS); both heme and Hb supplementation decreased the ZnMP uptake capacity by approximately 50% (Figure 3). These results suggested that membrane transport proteins involved in ZnMP uptake were induced under heme limitation conditions, as shown for *L*HR1 in *L. amazonensis* [14] and *L. infantum* [48].

### 3.7. L. major Promastigotes Efflux ZnMP

Despite its essential role, intracellular heme content must be tightly regulated since excessive heme levels lead to cell apoptosis by promoting oxidative stress and lipid peroxidation [49]. The only physiological mechanism identified for heme degradation in vertebrates is via the enzymatic action of the heme oxygenase protein, which requires NADPH and oxygen to degrade the heme group to iron, carbon monoxide, and biliverdin [50]. Additionally, transport proteins that mediate heme efflux to protect cells against oxidative stress induced by its accumulation have been described [51,52,53].

Although *Leishmania* can use heme as an iron source [20], the mechanism it uses to degrade the heme group is unknown. A previous study suggested the presence of heme oxygenase activity in *L. donovani* via direct detection of its enzymatic activity [54], whereas other authors reported that growth inhibition of axenic *L. infantum* amastigotes in iron-deficient medium is reversed by supplementation with Hb or hemin [20]. However, to date, no gene encoding a heme-oxygenase-like protein has been identified within the genomes of all *Leishmania* species sequenced. Since approximately 65% of the protein-coding genes in *Leishmania* spp. currently lack functional assignment, the presence of a heme-oxygenase-type gene, possibly differing significantly from its counterpart in other eukaryotic organisms, cannot be ruled out. On the other hand, putative heme efflux could be the only detoxification mechanism preventing the accumulation of toxic porphyrins inside the parasite.

**Figure 4:**
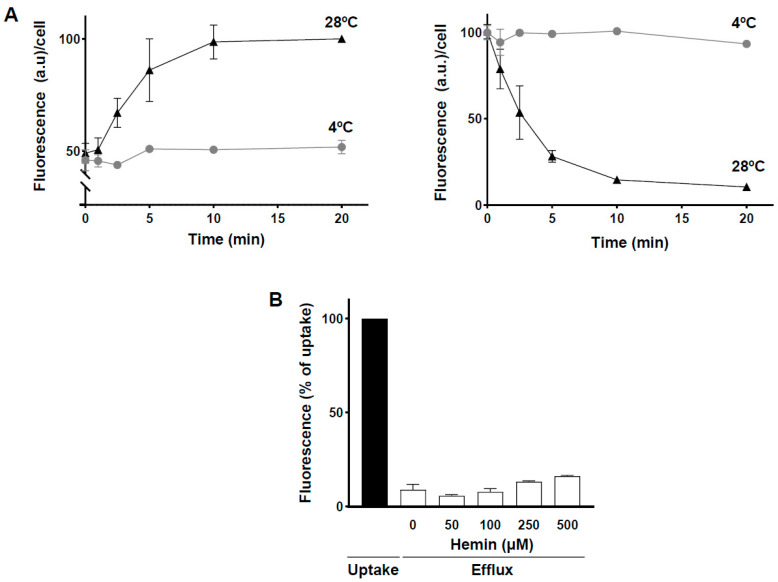
ZnMP efflux in *L. major* promastigotes. (**A**) ZnMP efflux. Parasites were pre-loaded with 10 µM ZnMP for 20 min at 28 °C, washed, and then incubated in medium without probe (HPMI glucose) at 28 °C (black) or 4 °C (gray) to determine the amount of fluorescent porphyrin retained and released from cells as a function of time. Intracellular ZnMP was measured via flow cytometry (left), while extracellular ZnMP (supernatant) was measured using spectrofluorimetry (right). (**B**) Effect of extracellular hemin on ZnMP efflux. Parasites were pre-loaded with 10 µM ZnMP and then incubated for 10 min in HPMI glucose medium containing increasing amounts (0–500 µM) of hemin at 28 °C (black) or 4 °C (gray). Intracellular ZnMP was quantified via flow cytometry as described above. The experiment was performed three times in duplicate and data expressed as mean ± SEM.

To study whether the parasites were capable of expelling porphyrins, *L. major* promastigotes were first cultured in the presence of the fluorescent heme analog, as described previously, then washed to remove non-internalized extracellular porphyrin. Subsequently, parasites were incubated without the probe at 28 °C or 4 °C. At the indicated times, intracellular porphyrin was measured via flow cytometry (Figure 4A left), whereas the extracellular probe was spectrophotometrically measured in the medium (Figure 4A right). As observed in Figure 4A, at 28 °C, there was a rapid efflux of intracellular ZnMP accompanied by an equivalent accumulation of the heme analogue in the extracellular medium, indicating that the decrease in intracellular fluorescence was not due to degradation of the probe. This ZnMP efflux was completely abolished at 4 °C.

ZnMP efflux was also studied in the presence of high concentrations of extracellular hemin. For this purpose, parasites were first incubated with the fluorescent probe and washed to remove non-internalized extracellular ZnMP as described above. Then, during the efflux process at 28 °C for 10 min, different concentrations of hemin (50–500 µM) were included in the extracellular medium. Flow cytometry analysis of the remaining intracellular porphyrin (Figure 4B) showed that efflux (0 µM hemin) is independent of the presence of extracellular hemin. Therefore, the ZnMP efflux mechanism is not inhibited by exogenous hemin and functions efficiently against a porphyrin concentration gradient.

This evidence suggested that heme efflux is an active process, possibly mediated by a transporter protein, although we cannot rule out a role for exocytosis. If a transporter is responsible for efflux, ABC family members could be candidate proteins for this function [41]—probably ones not inhibited by cyclosporin A and verapamil, which had no effect on porphyrin internalization (Figure 2D). Moreover, FLVCR family proteins have been shown to carry out porphyrin efflux in eukaryotic cells [51]. The only FLVCR protein described in *Leishmania* spp., (*L*FLVCRB) does not mediate heme efflux [15]. However, the parasite has three other FLVCR proteins of unknown function.

In conclusion, the presence of heme oxygenase in *Leishmania* cannot be ruled out. Nevertheless, promastigotes have a porphyrin detoxification mechanism consisting of its active efflux from the cell.

### 3.8. ZnMP Uptake Efficiency Differs between Leishmania Species and Does Not Correlate with LHR1 and LFLVCRB Expression Levels

The lower Km value obtained for porphyrin uptake in *L. major* (Figure 1D) compared to that previously reported in *L. donovani* [12] and the lower porphyrin uptake of *L. amazonensis* [14] compared to *L. donovani* [12] or *L. infantum* [48] suggested that heme uptake efficiency varies between *Leishmania* species. Indeed, *Leishmania* species amastigotes must be adapted to differential nutritional environments depending on cell tropism. Viscerotropic species, such as *L. donovani* and *L. infantum*, replicate in macrophages inside the liver and spleen—cells specialized in removing hemoglobin, heme, and senescent erythrocytes from circulation [55]. These processes are even increased in *Leishmania*-infected macrophages due to upregulation of the expression of the Hb scavenger receptor CD163 and downregulation of SIRPα (phagocytosis-inhibitory receptor) in response to parasite infection [56,57,58]. In this context, recycled hemoglobin and phagocyted erythrocytes within the infected macrophages provide an important source of heme (probably in the form of Hb) to support the parasites’ survival. On the other hand, *Leishmania* species responsible for cutaneous manifestations, such as *L. major*, predominantly infect dermis-resident macrophages, which play a role in promoting tissue homeostasis and repair instead of erythrophagocytosis or Hb scavenging [59]. Therefore, these parasites possibly have more limited access to heme, thus requiring a more efficient porphyrin uptake mechanism to fulfill their nutritional demands. Despite this, it was recently demonstrated that skin-residing CD163^+^CD91^+^ macrophages could phagocytose erythrocytes after a sand fly bite [60].

**Figure 5:**
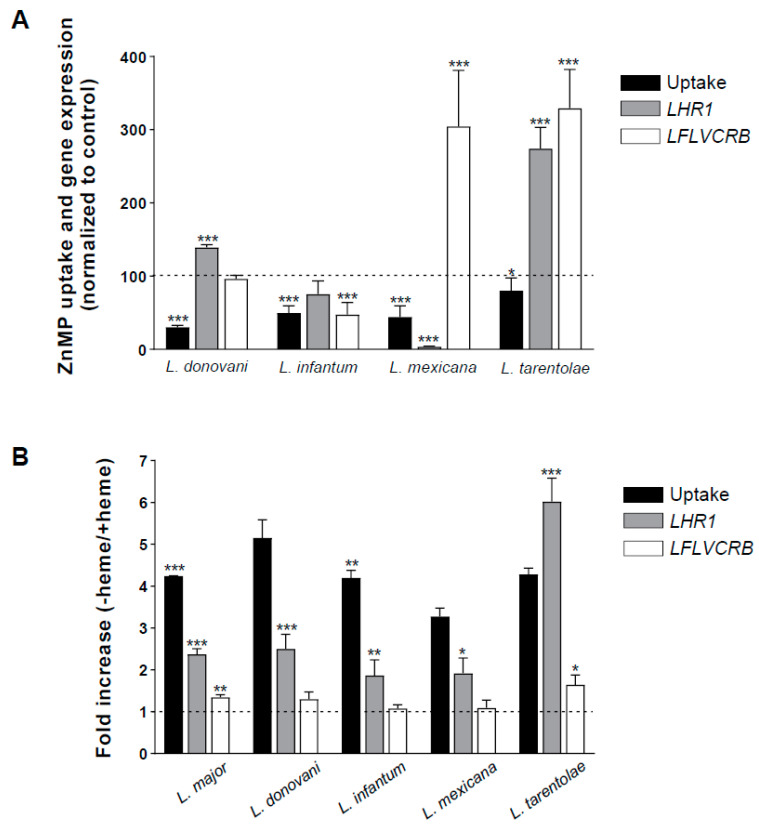
ZnMP uptake and heme transporters expression levels in different *Leishmania* spp. Promastigote forms of the indicated *Leishmania* species were cultured in 10% FBS-supplemented medium (**A**) or 20% hdFBS-supplemented medium containing or not 10 µM heme (**B**). Net ZnMP uptake was measured using flow cytometry after 10 min incubation with 10 µM ZnMP (black bars) as described in Materials and Methods. The *L*HR1 mRNA (gray bars) and *L*FLVCRB (white bars) expression levels were analyzed by qRT-PCR as described in Materials and Methods. Fold increase (−heme/+heme) represents the ratio of ZnMP uptake or gene expression in absence of heme relative to the presence of heme in the culture medium. Data in (**A**) were represented as percentage of the values found in *L. major* (dashed line). The experiment was performed three times in duplicate, and data expressed as mean ± SEM. *p* ≤ 0.05 (* *p* ≤ 0.05, ** *p* ≤ 0.005, *** *p* ≤ 0.0005) was considered statistically significant.In (**A**), significance analysis was performed comparing each value with that of *L. major*, whereas, in (**B**), it was performed comparing results with or without heme.

To analyze this point, we compared porphyrin internalization in different *Leishmania* species to determine whether these putative differences could be related to differences in the expression levels of the heme transporters *L*HR1 and *L*FLVCRB. We evaluated human species causing cutaneous (*L. major*), visceral (*L. donovani* and *L. infantum*), and cutaneous but also diffuse and mucocutaneous (*L. mexicana*) leishmaniasis together with the lizard-infecting species *L. tarentolae*, with *L. major* as the reference strain. First, ZnMP uptake was measured using promastigotes of the different species in their exponential growth phases grown in complete medium (including FBS). As shown in Figure 5A (black bars), the viscerotropic species *L. donovani* and *L. infantum* had significantly lower ZnMP uptake than *L. major* promastigotes (30% and 55%, respectively). However, *L. mexicana*, which produces dermic forms of leishmaniasis, also had a reduced uptake (50%) compared to *L. major* (Figure 5A). In addition, published results suggested that the viscerotropic *L. donovani* is more efficient at importing heme than *L. amazonensis*, which causes cutaneous disease [14,16]. In the case of *L. tarentolae*, ZnMP uptake is slightly lower than in *L. major* (20%) (Figure 5A). This species infects Old World geckos (not considered pathogenic to humans), where it predominantly colonizes lizard blood as free promastigotes [61]. Therefore, the efficiency of the process does not seem to be related to the parasite’s tropism but rather to each species. On the other hand, we cannot rule out that the scenario varies in amastigote forms of the parasite, which are those found in the parasitized mammal, since, for experimental reasons, the experiments shown and those previously published were carried out in promastigote forms of the parasite.

We used quantitative real-time PCR (qRT-PCR) to measure the expression levels of *L*HR1 (Figure 5A, gray bars) and *L*FLVCRB (Figure 5A, white bars) in promastigote parasites cultured under the same conditions (log phase parasites grown in medium with 10% FBS) and then analyzed the correlation with ZnMP uptake ability. Again, *L. major* was the reference strain. As shown in Figure 5A, whereas ZnMP internalization was reduced by 70% in *L. donovani* compared with *L. major*, the expression level of *L*HR1 increased by 40%, while *L*FLVCRB expression was similar. In *L. infantum*, the decreased ZnMP uptake (50%) was accompanied by a decrease in *L*HR1 expression, albeit not significant (25%; *p* = 0.224) and *L*FLVCRB (50%). For *L. mexicana*, with a 55% decreased ZnMP uptake compared to *L. major*, *L*HR1 expression was much lower (95%), while that of *L*FLVCRB was three-fold higher. Finally, in the case of *L. tarentolae*, the reduction in ZnMP uptake ability (20%) was accompanied by increased expression of *L*HR1 (2.7-fold) and *L*FLVCRB (3.3-fold). Therefore, there was no correlation between the ZnMP uptake capacity of the different species and their *L*HR1 and/or *L*FLVCRB expression levels.

Finally, we analyzed whether the increase in ZnMP uptake ability in the absence of heme was a general process in all these *Leishmania* species. We also studied the putative induction of *L*HR1 and *L*FLVCRB expression under these conditions. To normalize gene expression data, instead of the usual housekeeping gene *GADPH*, here we used a hypothetical protein (LmjF.04.0930) as the reference gene since its expression levels are not altered in response to heme, unlike *GADPH* in *Leishmania* (manuscript in preparation). Indeed, recent studies revealed that GAPDH is a heme chaperone that binds and transfers labile heme within mammalian cells [62], and a similar role could be carried out by *Leishmania* GAPDH. Thus, we cultured parasites in hdFBS-supplemented medium in the presence or absence of hemin before evaluating both their ability to capture ZnMP and their gene expression levels. Figure 5B shows that the induction of ZnMP uptake in the absence of heme (around 4–5-fold) was a general process in all *Leishmania* spp. analyzed. Similarly, induction of *L*HR1 expression in heme-depleted conditions was also observed in all species (around 1.8–2.5-fold) except in *L. tarentolae*, in which the induction level was higher (six-fold). Previously, Huynh et al. reported higher levels of *L*HR1 transcript induction (four-fold) in *L. amazonensis* promastigotes cultured in heme-deficient medium compared to heme-replete conditions [14]. For *L*FLVCRB, heme deprivation only slightly (10–65%) increased transporter expression, only achieving significance in *L. major* and *L. tarentolae*. The effect of heme deprivation on gene induction is lower than its effect on ZnMP uptake. However, the expression of these genes (especially *L*HR1) is clearly regulated by heme levels.

### 3.9. LHR1 and LFLVCRB Have Only Partially Overlapping Functions

Although *L*HR1 plays an additional role rescuing heme from endocytosed hemoglobin at its lysosomal location [12], both *L*HR1 and *L*FLVCRB share a common role at the plasma membrane importing heme [14,15,17]. In addition, abrogation of one *L*FLVCRB allele significantly increased *L*HR1 expression by 40%, and the deletion of one *L*HR1 allele also increased (≈three-fold) the abundance of *L*FLVCRB mRNA [15], again suggesting a functional overlap related to heme trafficking. However, both transporters are essential and affect parasite virulence [14,15]. To further explore a putative compensatory function between them, we used the CRISPR-Cas9 system in *L. major* to delete the *L*FLVCRB gene in parasites overexpressing the *L*HR1 gene from an episomal plasmid and vice versa. We found that *L*FLVCRB overexpression did not allow the elimination of the two *L*HR1 alleles, nor did *L*HR1 overexpression allow the generation of a double *L*FLVCRB KO (Table 1). As a control, this KO was easily obtained when the same gene was ectopically overexpressed from a plasmid (Table 1). Furthermore, it was not possible to delete both *L*FLVCRB or both *L*HR1 alleles in *L. major* parasites cultured in medium supplemented with a high level of an exogenous heme source either in the form of hemin or Hb. These results indicated that the functional overlap between *L*HR1 and *L*FLVCRB is not complete, suggesting that these proteins could play other essential functions in *Leishmania* parasites—for example, transporting another substrate in each case, as found for other MFS transporters such as HCP1, a proton-coupled folate transporter that can also transport heme [63]. This additional essential role of *L*HR1 and *L*FLVCRB besides heme transport together with the lack of correlation between heme uptake ability and *L*HR1 and *L*FLVCRB expression levels in different *Leishmania* species (Figure 5A), and the fact that the increased porphyrin import produced by *L*HR1 or *L*FLVCRB overexpression via episomal plasmids was much lower than that observed under heme depletion conditions [14,15] (Figure 3), means that we cannot rule out the putative existence of another functional, but as yet unidentified, porphyrin importer regulated by heme availability in the parasite.

On the other hand, *L*HR1, *L*FLVCRB and any other heme importer could be differentially expressed during the life cycle of the parasite, with overlapping substrate specificities in the case of heme but differential substrate affinities depending on pH, etc., leading to a heme uptake system with the capacity to adapt to a changing environment. Transcriptome profiling in *L. major* using RNA-seq has shown that, whereas *L*HR1 transcript levels were downregulated in metacyclic compared with promastigote parasites, *L*FLVCRB was upregulated [64]. In contrast, a significant increase in relative *L*HR1 mRNA levels in *L. major* was observed during the metacyclic promastigote to amastigote transition (four hours post-infection), whereas *L*FLVCRB transcript levels tended to decrease. In addition, *L. mexicana* showed a significant increase in both *L*HR1 and *L*FLVCRB mRNA levels during the 4 to 24 hpi transition [65].

## 4. Conclusions

All these results show that ZnMP uptake in *L. major* is an active, inducible and pH-dependent process not requiring a plasma membrane proton gradient but rather the presence of the monovalent cations Na^+^ and/or K^+^. In addition, this parasite is able to efflux this heme analogue against a concentration gradient. Furthermore, ZnMP uptake differs between different dermotropic or viscerotropic *Leishmania* species and does not correlate with the expression level of the heme transporters *L*HR1 and *L*FLVCRB, which have only partially overlapping functions. Altogether, these findings contribute toward a deeper understanding of an important process in the biology of these heme auxotrophic parasites responsible for important neglected diseases.

## Figures and Tables

**Table 1:** *L*HR1 and *L*FLVCRB are essential in *L. major* promastigotes. Attempts to delete both genes from the *L. major* genome using the CRISPR-Cas9ool kit [27]. Data show the number of clones of each genotype (wt, +/+; single knockout, +/−; double knockout −/−), analyzed as described in Material and Methods, obtained after transfection of parasites with or without an episomal copy of the indicated gene, or in cultured medium supplemented with 10 µM heme or 2.5 µM Hb.

	Genotype	-	Episomal *L*HR1	Episomal *L*FLVCRB	Heme	Hb
*L*HR1	+/+	0/24	0/24	0/11	0/24	0/24
	+/−	24/24	0/24	11/11	24/24	24/24
	−/−	0/24	24/24	0/11	0/24	0/24
*L*FLVCRB	+/+	0/24	0/24	0/24	0/20	0/24
	+/−	24/24	24/24	0/24	20/20	24/24
	−/−	0/24	0/24	24/24	0/20	0/24

## Supplementary Materials

The following supporting information can be downloaded at: https://www.mdpi.com/article/10.3390/ijms231810501/s1.
