# Supplementary material for: New Insights on Heme Uptake in Leishmania spp
Source: Int J Mol Sci. 2022 Sep 10;23(18):10501. doi: 10.3390/ijms231810501 (PMC9504327; doi:10.3390/ijms231810501)
Supplement: Supplementary file 1 [file ijms-23-10501-s001.zip › ijms-1802248-supplementary.pdf]

<u>Supporting information</u>

Supplementary Figure S1. *Emission spectrum of ZnMP as a function of pH*. The emission spectrum was obtained after excitation at 405 nm of a 10 µM solution of ZnMP at different pH.

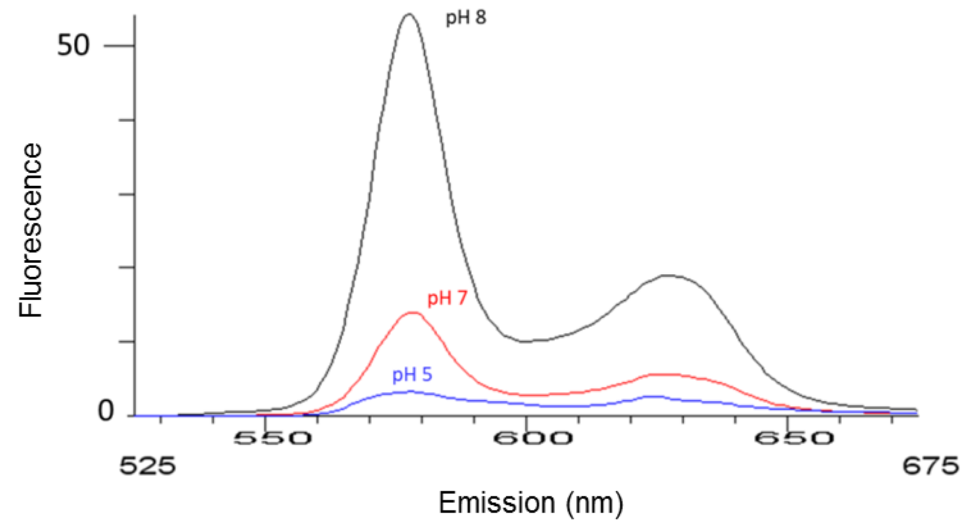

Supplementary Table S1. Oligonucleotides used.

**Primer sequence**	**Application**
TCTTGGTGACGCCGCAGTA	Forward primer to amplify *LHR1* from cDNA in *L. major*
GCCATCCACACGCCAGTC	Reverse primer to amplify *LHR1* from cDNA in *L. major*
TTTTGGTGCTGCCGATTGTG	Forward primer to amplify *LFLVCRB* from cDNA in *L. major*
CACTGCGGACACGATAAGG	Reverse primer to amplify *LFLVCRB* from cDNA in *L. major*
GCGGTGACGAGATGTTGACG	Forward primer to amplify *04.0930* from cDNA in *L. major*
GATTCCAGAAGCGGTGGCAG	Reverse primer to amplify *04.0930* from cDNA in *L. major*
TGAACGAGTTGGAGCGGAAG	Forward primer to amplify *LHR1* from cDNA in *L. donovani*
CAACAGAATCACGACGACGAAG	Reverse primer to amplify *LHR1* from cDNA in *L. donovani*
TTTTGGTGCTGCCGATTGTG	Forward primer to amplify *LFLVCRB* from cDNA in *L. donovani*
CACTGCGGACACGAGAAGG	Reverse primer to amplify *LFLVCRB* from cDNA in *L. donovani*
GCGGTGACGAGATGTTGACG	Forward primer to amplify *04.0930* from cDNA in *L. donovani*
GATTCCAGAAGCGGTGGCAG	Reverse primer to amplify *04.0930* from cDNA in *L. donovani*
TCTTTCTTTGCGCTTGCCTG	Forward primer to amplify *LHR1* from cDNA in *L. mexicana*
CGTCCTCTTCGAGTGTGTCC	Reverse primer to amplify *LHR1* from cDNA in *L. mexicana*
TTTTGGTGCTGCCGATTGTG	Forward primer to amplify *LFLVCRB* from cDNA in *L. mexicana*
CACTGCGGACACGATAAGG	Reverse primer to amplify *LFLVCRB* from cDNA in *L. mexicana*
GCGGTGACGAGATGATGACG	Forward primer to amplify *04.0930* from cDNA in *L. mexicana*
TGTGACGCACGACGCACTCT	Reverse primer to amplify *04.0930* from cDNA in *L. mexicana*
TGAACGAGTTGGAGCGGAAG	Forward primer to amplify *LHR1* from cDNA in *L. infantum*
CAACAGAATCACGACGACGAAG	Reverse primer to amplify *LHR1* from cDNA in *L. infantum*

Primer sequence	Application
TTGTGGTGCTGCCGATTGTG	Forward primer to amplify *LFLVCRB* from cDNA in *L. infantum*
CACTGCGGACACGAGAAGG	Reverse primer to amplify *LFLVCRB* from cDNA in *L. infantum*
GCGGTGACGAGATGTTGACG	Forward primer to amplify *04.0930* from cDNA in *L. infantum*
GATTCCAGAAGCGGTGGCAG	Reverse primer to amplify *04.0930* from cDNA in *L. infantum*
CTTCGCGGTTCTCTTAATCG	Forward primer to amplify *LHR1* from cDNA in *L. panamensis*
TTCGCTGTCCAGACTTGATG	Reverse primer to amplify *LHR1* from cDNA in *L. panamensis*
TACAAGAGCGAGAGAGGCAG	Forward primer to amplify *LFLVCRB* from cDNA in *L. panamensis*
ACAATAACGAAGCGAAACGG	Reverse primer to amplify *LFLVCRB* from cDNA in *L. panamensis*
GCGGGGATGAGATGATGACG	Forward primer to amplify *04.0930* from cDNA in *L. panamensis*
GATTCCAGAAGCGGTGGCAG	Reverse primer to amplify *04.0930* from cDNA in *L. panamensis*
GCATTCAGTATCGCCAGTCC	Forward primer to amplify *LHR1* from cDNA in *L. tarentolae*
CGCCACTTCATTGCTGCC	Reverse primer to amplify *LHR1* from cDNA in *L. tarentolae*
CCTCATCAAGGTATCCAGCAAC	Forward primer to amplify *LFLVCRB* from cDNA in *L. tarentolae*
CATCGCAGCCAGAACAATGG	Reverse primer to amplify *LFLVCRB* from cDNA in *L. tarentolae*
AGGCATCACCGGCATGAGCG	Forward primer to amplify *04.0930* from cDNA in *L. tarentolae*
CACGACGCACTCCCGCATGT	Reverse primer to amplify *04.0930* from cDNA in *L. tarentolae*

Supplementary Table S2. Accession number of the genes used.

	LHR1	*LFLVCRB*	Hypothetical protein
L. major	LmjF.24.2230	LmjF.17.1430	LmjF.04.0930
L. donovani	LdBPK_242320.1	LdBPK_171550.1	LdBPK_040930.1
L. infantum	LINF_240028500	LINF_170021800	LINF_040014300
L. mexicana	Lmx.24.2230	LmxM.17.1430	LmxM.04.0930
L. tarentolae	LtaP24.2390	LtaP17.1570	LtaP04.0900
